# Acute Recurrent Pancreatitis in a Pediatric Patient in the Setting of Viral Infection and COVID-19 Vaccination

**DOI:** 10.7759/cureus.40564

**Published:** 2023-06-17

**Authors:** Avery J Allen, Lindsey N Kudenchak

**Affiliations:** 1 Pediatrics, University of South Florida Morsani College of Medicine, Tampa, USA; 2 Pediatric Hospital Medicine, Lehigh Valley Health Network, Allentown, USA

**Keywords:** atypical presentation of covid-19, covid vaccination pancreatitis, covid-19 and pancreatitis, covid-19 vaccination, pediatric pancreatitis, pediatric gastroenterology, acute pancreatitis

## Abstract

Acute pancreatitis within pediatric populations is predominately caused by mechanical obstruction, trauma, medications, and infections. We present a case of an adolescent female without any known anatomic or metabolic pre-disposition, developing recurrent acute pancreatitis that is seemingly related to acute viral infection and COVID-19 vaccination.

## Introduction

Acute pancreatitis (AP) is estimated to affect over 11,000 pediatric patients annually [1]. However, with advances in imaging, diagnostic capabilities, and pathophysiological understanding, this figure is likely an underestimation. Common causes of AP in pediatric patients include infections, trauma, and mechanical obstruction [2]. Drug-induced pancreatitis has also been described [3]. The COVID-19 pandemic has accelerated the development and approval of mRNA vaccination, and according to the Centers for Disease Control and Prevention (CDC), it is not known to cause pancreatitis, though an association has been reported in adult patients [4]. Here, we present a case of an adolescent female who was diagnosed with AP on three distinct occasions, all of which were associated with either a presumed viral infection or the administration of the mRNA COVID-19 vaccination.

## Case presentation

**Figure 1:**
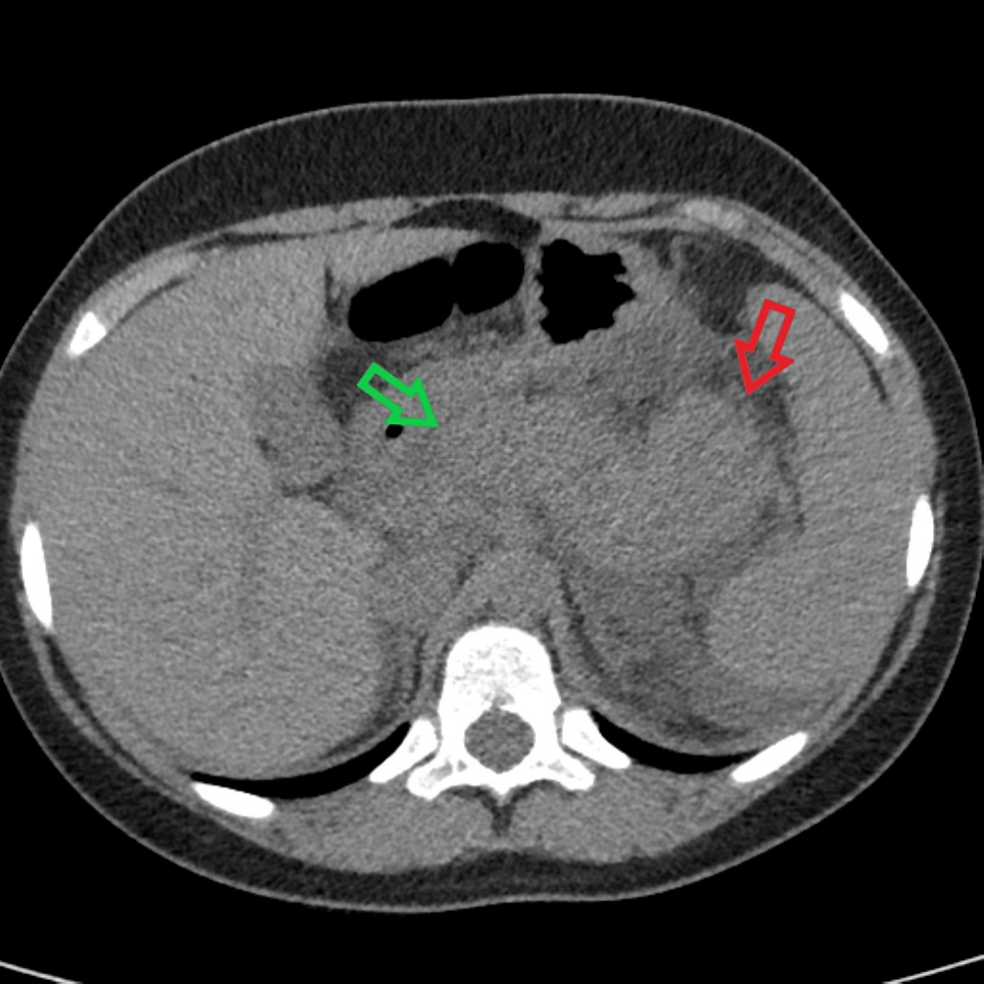
Non-contrast CT demonstrating the enlarged pancreas with extensive retroperitoneal and peripancreatic edema. An approximation of the head (green arrow) and the tail (red arrow) of the pancreas are shown. At the time of the study, it was noted that the lack of IV contrast limited the exam.

An 11-year-old female with no significant past medical history presented to the Emergency Department (ED) with complaints of constant, progressively worsening abdominal pain that began a few hours prior. She reported a sudden onset of symptoms with pain localized to the epigastric region and her back. She also admitted to some nausea and non-bloody, non-bilious vomiting. She denied any diarrhea, upper respiratory, or urinary symptoms. The only relevant findings on her physical exam were that she was mildly tender to palpation on her abdomen and peritoneal signs were absent. Her workup included a complete blood count (CBC), a comprehensive metabolic profile (CMP), a lipid panel, and lipase. Relevant reported results included leukocytosis and an elevated lipase of 11,405 U/L (reference range: 0-160 U/L) with no transaminitis. Her serum triglycerides were also within normal limits. An abdominal and pelvic computed tomography (CT) was ordered (Figure 1) and was significant for extensive retroperitoneal peripancreatic edema, consistent with AP with no evident pancreatic necrosis, abscess, or pseudocyst. There was also no evidence of biliary duct dilation or stone.

The patient was ultimately diagnosed with AP, given morphine for pain control and ondansetron for her nausea/vomiting, and placed on IV fluids before being transferred from the ED to an inpatient hospital setting for monitoring. She became inexplicably febrile (Tmax 103.3 F) on day 2 of the hospitalization. Blood cultures and urinalysis yielded no significant findings. No viral panels were obtained during her hospitalization as her fever was thought to be a sequela of pancreatitis. COVID-19 testing was not widely available at this time as this hospitalization was in February 2020. She was monitored as her fever resolved, lipase levels decreased, and pain improved. She was discharged home on day 6 with the diagnosis of idiopathic pancreatitis. 

Second admission

Seventeen months after the initial admission, the same patient, now 12 years of age, presented to the ED with a one-day history of mild abdominal pain. She reported that this pain started in the epigastric and periumbilical regions and had been waxing and waning throughout the past day. She denied any symptoms indicative of systemic infection and reported that this was the first occurrence of this type of pain since her discharge in 2020. She denied any recent infection, yet reported having received the Pfizer mRNA COVID vaccination 11 days prior.

Her CBC showed a white count elevation of 14,500 per centimeter cube (K/cm^3^) (reference range: 6.0-17.0 K/cm^3^) and a lipase elevation of 945 IU/L (reference range: 0-160 U/L). She had no transaminitis. She was diagnosed with recurrent AP and administered Pepcid, Maalox, and Toradol for gastritis and analgesia, respectively, along with IV fluids. When her symptoms resolved, she was discharged, and an outpatient appointment was scheduled with a pediatric gastroenterology (GI) specialist at a tertiary pediatric specialty center for further analysis of concerns regarding recurrent AP.

Three months after her discharge, she had a follow-up with the pediatric GI specialist regarding her “acute recurrent pancreatitis.” Magnetic resonance cholangiopancreatography was unremarkable. The specialist considered autoimmune causes, genetic predispositions, or celiac disease. Those tests, however, yielded no findings that would have predisposed her to recurrent episodes of pancreatitis. Her condition was ultimately classified as “idiopathic acute recurrent pancreatitis.” There was suspicion that COVID-19 infection could have been the inciting agent in her first bout, although diagnostic testing was not performed at the time (February 2020) due to technological shortcomings and/or the lack of knowledge about the virus.

Two years and nine months after her first hospitalization, the patient, now 13 years of age, presented to the ED with epigastric pain that radiated to the back, consistent with her past instances of pancreatitis. This instance occurred three days after receiving the Moderna mRNA COVID-19 booster immunization. Her home COVID test was negative. Her CMP was within normal limits, and lipase was 2,924 IU/L (reference range: 0-160 U/L). An ultrasound was completed to rule out biliary causes, which yielded no findings suggestive of alternative causes of the lipase elevation. The patient was diagnosed with recurrent AP and admitted to the floor for further observation and management. She was discharged and encouraged to follow up with her GI specialist.

## Discussion

AP in pediatric patients was previously thought to be a rather rare occurrence; however, recent evidence suggests that it has become more common, with incidences nearing 1/10,000 [5]. Up to 30% of reported cases are due to mechanical obstruction in the form of gallstones [2]. This is becoming a more prominent issue due to the obesity epidemic and the prevalence of elevated BMI in pediatric populations, which predisposes patients to develop gallstones [6]. This can theoretically predispose a patient to mechanical obstruction-induced pancreatitis. Drug-induced pancreatitis has also been reported and is thought to be among the other common causes of AP in pediatric patients, yet incidence is difficult to discern due to underreporting [3]. Trauma-induced pancreatitis represents another large portion of pediatric patients diagnosed with AP [7].

Recurrent cases in a pediatric patient with no anatomical or metabolic conditions that would predispose one to develop AP are undoubtedly abnormal. In pediatric patients, acute recurrent pancreatitis is defined as having AP on two distinct occasions and can predispose to the development of chronic pancreatitis [8]. In the case of this patient’s first bout with AP, a COVID-19 infection was not definitively ruled out. There are reports of MIS-C and COVID-19 infection resulting in AP in pediatric patients [9], and viral pancreatitis is among the common etiologies of AP in this demographic [5]. Observational studies in 2020 reported pancreatitis as the presenting symptom of COVID-19 in 1.25% of cases [10]. Due to the timeframe of her initial presentation, COVID-19 infection was never ruled out as a potential etiology for the first occurrence. Her first visit was prior to widespread quarantine, knowledge about the virus, or testing capabilities. 

COVID-19 vaccination has been proposed as a potential etiology of AP in both adult [4, 11] and adolescent [12] patients. Due to limited knowledge and the accelerated approval process of mRNA vaccines, it is not unreasonable to propose a potential association between the administration of an mRNA vaccine and AP in pediatric populations. The second instance of AP in this patient was reasonably close (within two weeks) to her initial mRNA-COVID-19 vaccination. This theory is strengthened by this patient’s third case of AP, which occurred after another mRNA booster was administered.

## Conclusions

It is obviously impossible to retroactively prove that this patent's first bout with AP was due to COVID-19, but the relation to subsequent vaccine administration, timing immediately prior to the global pandemic, and somewhat unsubstantiated symptoms is undoubtedly intriguing. Is it possible that her repeated bouts of pancreatitis represent an immunological phenomenon? Considering that antibodies are created via immune response to acute infections, as well as downstream of mRNA vaccination, could these downstream products be capable of manifesting clinically as AP? As literature has reported multiple instances of both COVID-19 infection and vaccination giving rise to pancreatitis, more research is needed to determine the mechanism of this link. Meanwhile, clinicians should consider COVID-19 infection and COVID-19 mRNA vaccination as potential etiologies in pediatric patients presenting with AP. 
